# Estimating Macroinvertebrate Biomass for Stream Ecosystem Assessments

**DOI:** 10.3390/ijerph19063240

**Published:** 2022-03-09

**Authors:** Kenneth W. Cummins, Margaret Wilzbach, Brigitte Kolouch, Richard Merritt

**Affiliations:** 1 Department of Entomology, Michigan State University, East Lansing, MI 48824, USA; kc8161@gmail.com (K.W.C.); merrittr@msu.edu (R.M.); 2 Department of Fisheries Biology, Humboldt State University, Arcata, CA 95524, USA; 3 Pymatuning Laboratory of Ecology, University of Pittsburg, Linesville, PA 16424, USA; brigitte@email.com

**Keywords:** functional feeding groups (FFGs), length–weight relationships of freshwater invertebrates, stream ecology, stream insects

## Abstract

We propose a field procedure for estimating the dry biomass of stream macroinvertebrates. Estimates are calculated using the mean values of the a and b regression coefficients from unpublished data and an extensive review of the relevant literature. The regression equation employed for calculating dry biomass is one that has been extensively used: Y = aX^b^, where Y = mg dry mass of an individual macroinvertebrate; X = mm total body length of an individual macroinvertebrate; a = intercept coefficient of the Y on X regression; and b = slope coefficient Y on X. The procedure was developed for use in the field, but dry mass estimates can also be made on preserved specimens. The case is made for presenting stream macroinvertebrate dry biomass data categorized by functional feeding groups (FFGs) and their component higher level taxa. The tables summarize the FFGs and their food resources, mean regression coefficients, dry biomass estimates for FFG-taxa by size and a comparison of their numerical-to-gravimetric surrogate FFG ratios to predict the stream environmental condition. A sizing template for rapidly sorting macroinvertebrates in the field is described. Thresholds for surrogate FFG ratios that directly predict measured stream ecosystem conditions are described.

## 1. Introduction

For decades, the taxonomic composition and relative abundance of macroinvertebrates have served worldwide as major tools for evaluating the environmental condition of running water ecosystems (e.g., [1,2,3,4,5]).

With few exceptions, stream studies have reported numerical macroinvertebrate data (e.g., [5]. However, when stream macroinvertebrate biomass equivalents are reported, the interpretation of the data relative to the stream ecosystem condition can be significantly different. For example, one terminal instar *Tipula* crane fly larva (Diptera, Tipulidae) would be approximately 14 times heavier than one terminal instar midge larva (Diptera, Chironomidae) (7.28 mg dry mass vs. 0.58 mg dry mass.

When presenting macroinvertebrate biomass data, a good case can be made for categorizing them as functional feeding groups (FFGs) (e.g., [6,7,8,9,10]). Additionally, the biomass ratios of the FFGs can serve as surrogates for directly measured stream ecosystem environmental attributes [7].

In this paper, we propose a simple procedure for estimating the dry biomass of the taxonomic components of macroinvertebrate functional feeding groups (FFGs) for use in a stream ecosystem or other analyses. The method can be readily accomplished in the field on live individuals or in the laboratory using preserved specimens. We first describe the procedure and then the basis for the procedure.

## 2. Macroinvertebrate Functional Feeding Groups (FFGs)

In the 1950s, the eminent freshwater invertebrate biologist Robert Pennak [11,12] held that stream ecosystem studies would only be valuable if macroinvertebrate taxonomy was at the species level. This was not realistic in the 1950s nor is it now. The fifth edition of “Aquatic Insects of North America” [13] essentially provides keys to every genus in North America but contains no species keys. Species keys for aquatic insects are limited to keys for specific genera or a restricted geographic area (see references in [13]). However, genus and species identification is valuable in many contexts [14]. The functional feeding group (FFG) approach was proposed in the 1970s [8,9,10,15] in response to the perceived taxonomic limitation of stream ecosystem studies using macroinvertebrates. The basic concept is that stream macroinvertebrate populations can be classified into six FFGs based on their adaptations for acquiring six categories of food resources (Table 1). The concept is applicable worldwide [6].

These relationships are: (1) scrapers (SC), which feed on attached non-filamentous single cell or colonial algae in riffles; (2) herbivore shredders (HSH), which feed on live, rooted aquatic vascular plants; (3) detrital shredders (DSH), which feed on leaf litter, of terrestrial riparian origin that has been microbially conditioned by aquatic hyphomycete fungi; (4) gathering collectors (GC), which feed on fine particulate organic matter (FPOM) deposited on or in stream bottom sediments; (5) filtering collectors, which feed on FPOM in suspension transported in the current; and (6) predators, which feed on live invertebrate prey (e.g., [7,16]).

## 3. Sorting, Measuring and Estimating FFG-Taxa Biomass in the Field

Although both numerical and biomass determinations are useful in stream ecosystem evaluations, the FFG-taxa approach provides the more rapid procedure of categorizing stream macroinvertebrates for estimating dry biomass in the field. Numerical taxonomic data are most useful in assessing the distribution and abundance of rare and at-risk species in water quality assessments. For example, the presence and densities of invertebrate taxa with univoltine vs. longer life cycles usually indicates a high water quality status (e.g., [4,17]). Most water quality studies and indices utilizing macroinvertebrates only report numerical taxonomic data (e.g., [18,19]). Numerical data also have been the first step in estimating macroinvertebrate production (e.g., [17,20,21,22]). 

By comparison, biomass data are fundamental to the measurement of ecosystem structure and dynamics, including carbon cycling, secondary production, and trophic structure [7]. In studies of energetics, biomass data are required for determining caloric values, either measured directly or estimated by conversion from tables of calories per unit biomass [23].

Because directly measured dry biomass data on stream macroinvertebrates are limited (e.g., [24,25]), biomass values are estimated by regression analyses. The regression used is: Y = aX^b^, where Y = mg dry biomass of an individual; X = mm total body length of that individual; and a and b are coefficients—a = the intercept of a Y on X; and b = the slope of Y on X [14,24,25,26,27]. The average coefficient values listed by FFG-taxa presented in Table 2 were used to calculate the dry biomass data in Table 3. If macroinvertebrate data are only to be given at the order level, regression coefficients can be found in Table 4, Table 5 or other literature.

Following the collection of macroinvertebrate samples from a site using standard sampling devices (e.g., a 30 s timed stream sample taken with a D-Frame net; or a sampler that samples a fixed area such as a Surber sampler), the first step is sorting the animals. Each sample is washed into a tray, and the animals are removed and sorted by FFG and taxonomic groups (e.g., Scrapers, Heptageniidae (Table 2)). An 8- or 12-well muffin tin is useful for keeping the FFG-taxa groups separate. The next step is to enumerate the number of individuals in each given FFG-taxa group into 5 mm size bins (e.g., ≤ 5 mm, > 5 ≤ 10, > 10 ≤ 15, etc.). This can be accomplished using a template similar to that provided in the inside back cover of Merritt et al. (2019) [13]. The template illustrates a series of nine circles, with each circle increasing in diameter by 5 mm increments—5, 10, 15 mm, etc., until 45 mm. A transparency copy of the circles can be made to fit the bottom of the sorting tray. Each invertebrate in the FFG-taxa being measured is moved into the circle in which it fits best. The number of individuals in each 5 mm circle is recorded for each FFG-taxa group and entered into a data sheet. The size of the individuals that fit in any 5 mm increment circle represents a range. For example, the 5 mm circle can contain individuals ranging in length from 0.1 to 5.0 mm, the 10 mm circle contains animals ranging in length from 5.1 to 10 mm, etc. The midpoint of the size range for each circle has been used as the value of X in the regression to estimate the biomass values listed by the size bin in Table 3. The number of individuals in a circle is then multiplied by the estimate of biomass per individual for that size group to arrive at the estimate of the total biomass of the FFG-taxa of that size. For example, if there are seven individuals in the Ephemeroptera, of the Heptageniidae category of scrapers in the 15 mm diameter circle, then the biomass for that FFG-taxa group would be 28.86 * 7 = 202.02 mg. The sum of the biomass from all the circles is equal to the estimated total biomass of that FFG-taxa group. Finally, the total of all the biomass estimated for each FFG is recorded for use in calculating the FFG surrogate ratios (Table 4).

## 4. FFG Ratios as Surrogates for Directly Measured Stream Ecosystem Attributes

The direct measurement of stream ecosystem environmental conditions is time consuming and labor intensive. If samples or measurements are made with an automated recording device, the dataset will be limited in time and space. An advantage of using macroinvertebrates to assess stream ecosystem conditions is that these integrate space and time conditions in a stream reach by their presence and relative abundance during the period of their life cycle spent in the stream [7,17]. The presence and abundance of macroinvertebrate FFGs reflect the availability of respective food resources; in turn, the abundance and availability of these food resources depend on stream ecosystem conditions (Table 1). For scrapers and herbivorous shredders, critical conditions include the levels of light and nutrients. For detritivorous shredders, the characteristics of the stream-side riparian vegetation and the availability of microbes to condition plant litter in oxygenated habitats after the litter enters the stream are important. The availability of fine particulate organic matter (FPOM) on or in the stream sediments affects the abundance of gathering collectors, and filtering collectors depend on the availability of FPOM transported in the current (FC). Total predators are compared to the total of all other FFGs (potential live prey) to assess the potential stability of the predator–prey balance. A situation in which the total predator biomass, estimated at a point in time, is equal or greater than that of the live prey can be sustained only if the turnover of the prey biomass (generation time, i.e., from egg to adult) exceeds that of their predators. The majority of stream macroinvertebrate predators are larger and have longer generation times than their prey, and thus a slower turnover time. Some prey are univoltine (one generation per year), but most are polyvoltine (two or more generations per year); most predators have univoltine or longer generation times [17].

Some surrogate FFG ratios have been compared to the direct measures of the stream ecosystem attributes that they predict. For example, the functional group composition was compared with the measurements of the primary production and community respiration determined using respiration chambers [67,68]. However, in most instances, stream ecosystem conditions have been based on qualitative assessments [5,69,70,71,72] in which thresholds for assigning a value to a particular ecosystem attribute are presentedwith specific threshold values which differ depending on the seasonality of the sampling [9]. In Table 4, we present examples of ecosystem assessments based on FFG ratios in two coastal streams in northern California. The stream ecosystem attribute P/R is the ratio of gross primary production to total community respiration. For the reach of a stream ecosystem, this allows it to be characterized as autotrophic vs heterotrophic, which is arguably the most fundamental measure of a stream ecosystem’s condition [73]. Autotrophic streams are driven by algal and rooted aquatic vascular plant production as their primary energy source. By comparison, heterotrophic stream reaches are dependent on terrestrial plant litter from the riparian zone as their dominant energy source. A directly measured P/R ratio > 1.0 indicates autotrophy. A corresponding FFG ratio of autotrophic dominance is 0.75 (Table 4, [8,67,68]).

Because the surrogate FFG ratios are dimensionless numbers, they are relatively independent of the sample size. For example, the FFG calculated from one subsample from a given stream habitat such as a riffle was found to be statistically similar to the average of additional samples (Cummins, unpublished).

Our intent was to present a scenario that uses aquatic macroinvertebrates to characterize the environmental condition of a stream reach. We propose a sequence of macroinvertebrate sampling, sorting and identification to FFG or major morphogical categories within FFG, rapidly measuring body lengths, and the use of published regression analyses to estimate biomass, to finally arrive at FFG ratios that are able to accurately characterize the environmental conditions of a stream ecosystem. Extensive validation of the procedure will undoubtedly lead to significant advances.

## Figures and Tables

**Table 1:** Stream macroinvertebrate functional feeding groups (FFGs) and the corresponding food resource categories upon which they depend.

Functional Feeding Group (FFG)	Food Resource Category (FRC)
Scrapers (SC)	Attached non-filamentous algae (especially diatoms)
Herbivore shredders (HSH)	Rooted aquatic vascular plants
Detrital shredders (DSH)	Leaf litter of riparian origin conditioned by hyphomycete fungi (coarse particulate organic matter or CPOM)
Gathering collectors (GC)	Fine particulate organic matter on or in the bottom sediments (benthic fine particulate organic matter or BFPOM)
Filtering collectors (FC)	Fine particulate organic matter in transport in the water column in the current (TFPOM)
Predators (P)	Live invertebrate prey

**Table 2:** Intercept and slope coefficients used in the regression equation Y = aX^b^ where Y = dry biomass in mg; X = total body length in mm; a = intercept of Y on X; and b = slope of Y on X, organized by the macroinvertebrate taxa and functional feeding groups (FFGs) of stream macroinvertebrates. Coefficients were averaged from regressions used in the study by Cummins (unpublished) and from the representative literature. All insect body lengths, in mm, were measured from the front of the head to the end of abdomen, excluding filaments and head appendages: for Oligochaeta, L = mm body length; Mollusca, Gastropoda H = shell height; Mollusca, Bivalvia W = shell width, H = shell height.

Higher Taxa	Morphology and Behavior Characteristics	FFG (Functional Feeding Group)	Coefficients and Number of Studies (n)	Taxonomic Source of Coefficients	References
Oligochaeta (segmented worms)	Long slender, round in cross-section, 2 lateral chaetae on both sides of each segment	Gathering collector (GC) (Y = aL, L = length)	a = 0.3657	Oligochaeta	Cummins (unpublished)
Crustacea (scuds amphipods)	Flat side to side, more than 6 legs, arched dorsal line of back with posterior directed spines on each segment	Scrapers (SC)	a = 0.0037 b = 3.003 *n* = 4	Amphipoda: Gammaridae, *Hyallela*	[24,28,29,30]
Crustacea (side swimmers amphipods)	Flat side to side, more than 6 legs, arched dorsal line of back smooth	Detrital shredders (DSH)	a = 0.0032 b = 2.948 *n* = 2	Amphipoda: Gammaridae, *Gammarus*	[24]
Crustacea (sow bugs)	Oval shape in dorsal view, more than 6 legs, flat top to bottom	Detrital shredders (DSH)	a = 0.0032 b = 2.948 *n* = 2	Isopoda: Asellidae, *Asellus*	[24]
Mollusca (snails)	Spiral-shaped shells, height greater than width, less in flat-shaped Ancylidae, retractable muscular foot	Scrapers (SC)	a = 0.0269 b = 3.003; *n* = 17 (Y = aH^b^, H = Shell height)	Gastropoda: Physidae, Pleuroceridae, Ancylidae	[24,31,32]
Mollusca (clams)	Oval shells in side view, flat to round side to side, incurrent and excurrent siphons	Filtering collectors (FC)	a = 0.0435 b = 2.637 *n* = 7 Y = aW^b^, W = shell width	Bivalvia (=Pelecypoda): Sphaeridae, Unionidae	[24,33,34,35,36]
Crustacea (crayfish)	Long oval shape in dorsal view, shallow arched in cross-section, first appendages large claws	Detrital shredders (DSH)	a = 0.0098 b = 3.347 *n* = 6	Decapoda: *Orconectes, Cambarus*	[24,37,38,39]
Ephemeroptera (riffle mayflies)	3 (or 2) terminal long filaments, lateral abdominal gills, flat body cross-section	Scrapers (SC)	a = 0.0072 b = 2.659 *n* = 20	Heptageniidae, some Ephemerellidae, *Drunella*, Ameletidae	Cummins (unpublished), [24,25,40,41,42,43,44,45]
Ephemeroptera (sprawling and swimming mayflies)	2 (or 3) terminal filaments, lateral abdominal gills, oval body cross-section	Gathering collectors (GC)	a = 0.0057 b = 2.966 *n* = 9	Baetidae, Leptophlebiidae, Ephemerellidae (not *Drunella*). Caenidae, Siphlonuridae	Cummins (unpublished), [24,25,41,42,43,46,47,48,49,50]
Ephemeroptera (clinging filtering mayflies)	3 terminal long filaments, inside of front legs with long hairs used for filtering	Filtering collectors (FC)	a = 0.0105 b = 2.820 *n* = 3	Isonychidae	[24,25,47,51,52]
Plecoptera (predator stoneflies)	2 terminal filaments, no lateral abdominal gills, color pattern, large eyes, very active	Predators (P)	a = 0.0131 b = 2.606 *n* = 23	Perlidae, Perlodidae, Chloroperlidae	Cummins (unpublished), [24,42,53,54]
Plecoptera (detritivore stoneflies)	2 terminal filaments, no lateral abdominal gills, large or small roach-like, brown, legs and underside lighter, small eyes, sluggish	Detrital shredders (DSH)	a = 0.0140 b = 2.700 *n* = 9	Pteronarcyidae, Taeniopterygidae (large). Peltoperlidae (small roach-like)	Cummins (unpublished), [42,53,54]
Plecoptera (detritivore stoneflies)	2 terminal filaments, no lateral abdominal gills, small, slender, uniform black/brown, small eyes, sluggish	Detrital shredders (DSH)	a = 0.0046 b = 2.676 *n* = 11	Nemouridae, Capniidae, Leuctridae	[24,25,42,49,55]
Trichoptera (small scraper caddisflies)	Small with mineral non-tapered case, may have lateral balance stones, larvae with small terminal lateral hooks	Scrapers (SC)	a = 0.0070 b = 2.410 *n* = 4	Glossosomatidae, Helicopsychidae, Goeridae, Turenmatidae, some Limnephilidae (without lateral balance sticks)	Cummins (unpublished), [24,56,57]
Trichoptera (detrital shredder caddisflies)	Organic non-tapered case, large with lateral balance sticks, or small without lateral sticks, larvae with small terminal lateral hooks	Detrital shredders (DSH)	a = 0.0033 b = 2.660 *n* = 8	Most Limnephilidae, Calamoceratidae, Lepidostomatidae	Cummins (unpublished), [24,56]
Trichoptera (gathering collector caddisflies)	Mineral and/or organic tapered or cone-shaped case, slender larvae, with small terminal lateral hooks	Gathering collectors (GC)	a = 0.0083 b = 2.149 *n* = 5	Leptoceridae, Odontoceridae	Cummins (unpublished), [24,56]
Trichoptera (net spinning caddisflies)	Fixed retreat with capture net, larvae with long curved ventrally oriented curved hooks	Filtering collectors (FC)	A0.0038 B = 3.610 *n* =18	Hydropsychidae, Philopotamidae, Polycentropodidae	Cummins (unpublished), [24,25]
Trichoptera (predator caddisflies)	Large active free living (pupa case only) larvae with stout head, long curved ventrally oriented curved hooks	Predators (P)	a = 0.0050 b = 3.083 *n* = 2	Rhyacophilidae	Cummins (unpublished), [24,25]
Coleoptera (predator beetle larvae)	Oval cross-section, large mandibles, lateral abdominal projections	Predators (P)	a = 0.0013 b = 3.300 *n* = 2	Dytiscidae, Hydrophilidae	[24]
Coleoptera (water penny beetle larvae)	Flat disc-shaped larvae, body concealed beneath broad shield of dorsal plates	Scrapers (SC)	a = 0.0123 b = 2.906 *n* = 1	Psephenidae	[24]
Coleoptera (riffle beetle larvae)	Arched cross-section retractile gills in ventral terminal posterior abdominal chamber	Gathering collectors (GC)	a = 0.0079 b = 2.879 *n* = 2	Elmidae	[24]
Coleoptera (predaceous diving beetle adults)	Hard shell elytra, hind legs modified for swimming, antennae longer than labial palps	Predators (P)	a = 0.0420 b = 2.657 *n* = 3	Dytiscidae	[24]
Coleoptera (water scavenger adult beetles)	Hard shell elytra, hind legs modified for swimming, labial palps longer than antennae	Gathering collectors (GC)	a = 0.0473 b = 2.611 *n* = 1	Hydrophilidae	Cummins (unpublished)
Coleoptera (riffle beetle adults)	Very long crawling legs	Scrapers (SC)	a = 0.0474 b = 2.681 *n* =2	Elmidae	[24]
Megaloptera (Dobsonfly larvae)	Lateral abdominal filament projections, terminal caudal hooks	Predators (P)	a = 0.0045 b = 2.935 *n* = 6	Corydalidae	Cummins (unpublished), [24,58]
Megaloptera (alderfly larvae)	Lateral abdominal filament projections, single, long terminal abdominal filament	Predators (P)	a = 0.0037 b = 2.753 *n* = 2	Sialidae	[24]
Lepidoptera (aquatic moth larvae)	Pair of abdominal prolegs on each abdominal segment with terminal semicircle of tiny hooks (crochets)	Herbivore shredders (HSH)	a = 0.0020 b = 2.807 *n*= 2	Crambidae, Noctuidae	[24,59]
Odonata (dragonfly nymphs)	Long extendible grasping labium, terminal abdominal caudal spines	Predators (P)	a = 0.0086 b = 2.821 *n* = 10	Anisoptera:Aeschnidae, Libellulidae, Gomphidae, Cordulegastridae	[24]
Odonata (damselfly nymphs)	Long extendible grasping labium, 3 terminal paddle-shaped caudal gills	Predators (P)	a = 0.0048 b = 3.256 *n* = 4	Zygoptera: Agrionidae, Coenagrionidae	[25]
Hemiptera (predaceous nymph and adult water bugs)	Long pointed piercing beak, oval in dorsal view	Predators (P)	a = 0.0234 b = 2.637 *n* = 3	Belostomatidae, Veliidae, Gerridae)	[25]
Hemiptera (water boatman nymph adult)	Short triangular beak, longer than wide in dorsa view	Scrapers (SC)	a = 0.0234 b = 2.637 *n* = 3	Corixidae	[25]
Diptera (black flies, with biting adults)	Bowling pin-shaped, complete head capsule with filtering head fans tiny hooks a tip of abdomen	Filtering collectors (FC)	a = 0.0027 b = 3.084 *n* = 8	Simuliidae	[24,60,61,62]
Diptera (Chironomidae, non-biting adult midges)	Large midge larvae, head capsule complete prolegs behind head, quadrate head equal to body width	Predators (P)	a = 0.0019 b = 2.614 *n* = 6	Tanypodinae	[24,25,63]
Diptera (Chironominae, filtering midges)	Small round head with long antennae, erect pronged tube with silk strands strung on the prongs	Filtering collectors	a = 0.0009 2.257 *n* = 3	Chironominae: Tanytarsini	[24,64]
Diptera (Chironomini, red and other midges)	Small round or longer head with very short antennae, burrowers in soft sediments	Gathering collectors (GC)	a =0.0023 b = 2.740 *n* = 14	Chironominae: Chironomini Orthocladiinae, Diamesinae	[24,46,64,65,66]
Diptera, Ceratopogonidae (‘no-see-um’ adult biting flies)	Very long needle-shaped, complete head capsule	Predators (P)	a = 0.0027 b = 2.439 *n* =3	Ceratopogonidae	Cummins (unpublished), [24,25]
Diptera, Tipulidae (crane flies)	Large robust larvae with only creeping welts, 2 terminal spiracular discs with surrounding lobes, incomplete head capsule	Detrital shredders (DSH)	a = 0.0031 b = 2.978 *n* = 53	Tipulidae, *Tipula, Lipsothrix*	Cummins (unpublished), [24,25]
Diptera (predaceous crane fly larvae)	Medium size slender larvae without prolegs, incomplete head capsule	Predators (P)	a = 0.0043 b = 2.632 *n* =8	Tipulidae (but not *Tipula*), Empedidae, Tabanidae	[24]

**Table 3:** Estimated dry mass per individual (mg) within length bins of 5 mm increments for each taxonomic category within its FFG. Estimates are based on averaged length–mass regression from the literature and the unpublished data of Cummins, at the midpoint of each size bin (e.g., at 7.5 mm for the >5 ≤ 10 mm bin). FFG = functional feeding groups; SC = scrapers; DSH = detrital shredders; GC = gathering collectors; FC = filtering collectors; P = predators; and HSH = herbivore shredders. Estimates are based on the relationship W = aL^b^, where W = dry biomass in mg; L = length in mm; and a = W intercept and b = slope of the relationship of W on L. Oligochaeta biomass based on constant (a) times the total length (L) of all worm segments in a sample as W: aL. Blank spaces indicate that the estimated dry biomass is out of the normal size range based on the literature.

FFG	Taxa	Morphology and/or Behavior	Dry Biomass mg (or g) by mm Length Size Groups								
			5	10	15	20	25	30	35	40	45
SC	MAYFLIES Heptageniidae *Drunella*	Wide, flat x-section nymphs, width >3 x height	0.96	7.58	28.86	64.35	126.73	Out of size range			
	CADDISFLIES Limnephilidae, Uenoidae, Glossosomatidae, Helicopsychidae, Odontoceridae	Stone case, stout larvae	5.40	48.32	174.23	432.81	876.64)	1560.53 (1.56 g)	2541.12 (2.54 g)	Out of size range	
	BEETLES, WATERPENNIES Psephenidae	Flat disc-like	0.50	6.12	26.48	74.84	Out of size range				
	BEETLES, RIFFLE ADULTS Elmidae	Long crawling legs	3.12	19.88	Out of size range						
	TRUE BUGS Corixidae	Stout triangular beak	1.63	10.14	29.55	Out of size range					
	SNAILS and LIMPETS *Physa, Juga, Ferrissia*	Spiral- or dome-shaped shells	2.95	18.06	59.63	139.24	268.76	459.95)	724.43	42,949.4 (42.95 g)	1519.31 (1.52 g)
DSH	STONEFLIES, LARGE Pteronarcyidae, Peltoperlidae	Uniform brown or black, sluggish	1.42	9.95	31.12	69.90	130.95	218.69	337.41	491.23	Out of size range
	STONEFLIES, SMALL Nemouridae, Capniidae, Leuctridae		0.63	4.46	14.00	31.54	Out of size range				
	CADDISFLIES Limnephilidae, Lepidostomatidae	Organic case, stout larvae	5.40	48.32	174.22	432.81	876.64	1560.53 (1.56 g)	2541.12 (2.54 g)	Out of size range	
	FLIES, CRANE FLIES *Tipula*	Incomplete head capsule, creeping welts, spiracular disc	0.21	1.41	4.28	9.40	17.31	28.50	43.45	62.60	Out of size range
	SCUDS *Gammarus*	Multiple legs, flat oval x-section	0.46	3.73	12.59	Out of size range					
	CRAYFISH	Multiple legs, round oval x-section, large claws	2.14	21.79	80.53	221.70	467.87	861.29	1442.85 (1.44 g)	2255.91 (2.26 g)	33,460.19 (3.35 g)
GC	MAYFLIES Baetidae, Caenidae, Ephemerellidae, Leptophlebiidae	Slender round x-section width and height equal	0.73	5.95	20.25	48.27	94.68	Out of size range			
	RIFFLE BEETLE LARVAE Elmidae	Small mandibles, x-section triangular to arched	0.59	5.43	19.81	49.62	Out of size range				
	FLIES, MIDGES Chironomini, Orthocladiinae	Round head capsule, single proleg under head	0.20	1.36	4.14	9.10	Out of size range				
	WORMS Oligochaeta	Segmented, no legs, 2 chaetae each side of each segment	1.83	3.66	5.49	7.31	9.14	10.97	12.80	14.63	18.29
FC	CADDISFLIES Hydropsychidae, Philopotamidae, Polycentropidae, Psychomyiidae	Capture net, long ventrally directed anal hooks	0.43	3.00	9.29	20.71	Out of size range				
	TRUE FLIES Tanytarsini	Case with prongs supporting capture net	0.06	0.52	0.75	Out of size range					
	Blackflies	Filtering head fans	0.39	3.28	11.44	Out of size range					
	CLAMS	Bivalve hard shell	18.86	117.30	211.28	513.04	779.98	1314.24 (1.31 g)	Out of size range		
P	STONEFLIES Perlidae, Perlodidae	Color pattern, large eyes	0.51	2.33	5.65	10.59	17.23	25.66	Out of size range		
	BEETLE LARVAE Dytiscidae Hydrophilidae	Oval x-section, large mandibles	1.92	29.04	143.29	439.24	Out of size range				
	BEETLE ADULTS Dytiscidae, Gyrinidae	Hard shell elytra, short palps	3.61	24.46	74.89	Out of size range					
	CADISS FLIES Rhyacophilidae	Free living, long caudal hooks	0.43	3.00	9.29	20.71	Out of size range				
	DOBSONFLIES Corydalidae	Lateral filaments, caudal hooks	0.28	1.47	3.60	31.72	60.22	101.67	Out of size range		
	ALDERFLIES Sialidae	Lateral filaments and terminal caudal filament	0.19	1.76	6.57	16.72	34.49	62.34	Out of size range		
	DRAGONFLIES Aeschnidae, Gomphidae, Cordulegastridae, Libellulidae	Extendible labium, caudal spines	0.65	4.63	14.48	35.54	61.03	101.83	157.13	228.80	318.71
	DAMSELFLIES Agrionidae, Coenagrionidae, Lestidae	Extendible labium, 3 plate-like caudal gills	0.39	3.14	10.61	25.17	49.22	85.14	135.30	Out of size range	
	TRUE BUGS Belastomatidae, Gerridae	Pointed beak, fore- wings half membranous	1.63	10.14	29.55	63.09	113.65	183.82	276.01	392.51	535.47
HSH	MOTHS Crambidae. Noctuidae	Ventral and caudal prolegs with crochets	0.29	2.47	8.66	21.08	42.02	73.83	118.93	179.72	258.30

**Table 4:** Functional feeding group (FFG) ratios as surrogates for the stream ecosystem attributes in two streams in coastal northern California, USA: Prairie Creek and Jacoby Creek, from the unpublished data of Wilzbach and Cummins. If the biomass assessment differs from the numerical assessment, then the biomass is italicized. PR = gross primary production/community respiration; SC = scrapers; SH = shredders; GC = gathering collectors; FC = filtering collectors; P = predators; CPOM = coarse particulate organic matter (>1 mm); and FPOM = fine particulate organic matter (>1 mm). Threshold values are from [7].

Ecosystem Parameter	FFG Ratio	Stream	Threshold	Number	Assessment	Biomass	Assessment
Autotrophy vs. heterotrophy	SC to SH + GC = FC	Prairie	>0.75	0.86	Autotrophic	0.54	*Heterotrophic*
		Jacoby		0.11	Heterotrophic	0.33	Heterotrophic
Availability of CPOM to FPOM	SH to GC + FC	Prairie	>0.50	0.15	Sparse food for shredders	0.04	Sparse food for shredders
		Jacoby		0.16	Sparse food for shredders	1.95	Abundant food for Shredders
FPOM in transport availability	FC to GC	Prairie	>0.50	0.14	Low food for filtering collectors	0.28	Low food for filtering collectors
		Jacoby		0.24	Low food for filtering collectors	0.05	Low food for filtering collectors
Stream bottom stability	SC + FC to SH + GC	Prairie	>0.50	1.09	Stable bottom dominates	0.95	Stable bottom dominates
		Jacoby		0.32	Stable bottom limiting	0.35	Stable bottom limiting
Predator to prey balance	P to SC = SH = FC = GC	0.17	0.10–0.15	0.17	High predator abundance	0.26	High predator abundance
		0.06		0.06	Low predator abundance	0.12	Normal predator abundance

**Table 5:** Regression coefficients for representative Ephemeroptera, Plecoptera, Trichoptera (EPT), based on stream collections made in Michigan, Pennsylvania, Idaho, Oregon, and Maryland, USA, from the unpublished data of Cummins. Coefficients a and b are based on regressions for each group. Sample sizes ranged from 39 to 100 individual aquatic insects that included a range of sizes. Mean coefficients were calculated from the plots of Y (dry mg) on X (total body length; a = intercept of Y on X and b = slope of Y on X. To convert any numerical EPT sample data to its dry biomass equivalent, multiply each taxonomic entry by thee total body length per individual times the number of individuals in the category and sum them.

Taxonomic Group	Coefficient a (Intercept)	Coefficient b (Slope)
Order Ephemeroptera (mayfly nymphs)		
Heptageniidae	0.00386	3.253
*Stenonema, Stenacron*	0.00339	3.320
Ephemerellidae, *Drunella*	0.001645	3.457
Order Plecoptera (stonefly nymphs)		
Perlidae	0.00430	3.061
Perlodidae	0.00281	3.036
Taeniopterygidae	0.00251	3.045
Order Trichoptera (caddisfly larvae)		
Glossosomatidae	0.00689	2.958
Limnephilidae, *Dicosmoecus*	0.00230	3.079
Lepidostomatidae	0.00293	#.243

## Data Availability

Data are contained within this article. Supplemental data are available from the authors on request.
